# Interzeolite Transformation from FAU-to-EDI Type of Zeolite

**DOI:** 10.3390/molecules29081744

**Published:** 2024-04-11

**Authors:** Stanislav Ferdov

**Affiliations:** Physics Centre of Minho and Porto Universities (CF-UM-UP), University of Minho, 4800-058 Guimarães, Portugal; sferdov@fisica.uminho.pt

**Keywords:** interzeolite transformation, hierarchical, FAU, EDI, room-temperature synthesis, OSDA-free synthesis

## Abstract

This study reports for the first time the transformation of the pre-made **FAU** type of zeolite to the **EDI** type of zeolite. The concentration of the KOH solution controls this interzeolite transformation, which unusually occurs at both room temperature and under hydrothermal conditions. The transformation involves the amorphization and partial dissolution of the parent **FAU** phase, followed by the crystallization of **EDI** zeolite. At room temperature, the transformation (11–35 days) provides access to well-shaped nano-sized crystals and hollow hierarchical particles while the hydrothermal synthesis results in faster crystallization (6–27 h). These findings reveal an example of an interzeolite transformation to a potassium zeolite that lacks common composite building units with the parent zeolite phase. Finally, this work also demonstrates the first room-temperature synthesis of **EDI** zeolite from a gel precursor.

## 1. Introduction

Kinetically, the zeolites are metastable phases generally transforming from low-density to high-density frameworks. This transformation is commonly observed during traditional zeolite synthesis, where an amorphous gel converts into a crystalline phase that transforms into another structurally distinct phase at longer crystallization times. Alternatively, precrystallized zeolites (parent phase) treated in a solution of alkali, alkali earth cations, and/or an organic structure-directing agent (OSDA) tend to transform to a different framework (daughter phase) and constitute a synthesis method known as interzeolite transformation (IZT) [1,2]. IZT offers a range of compelling benefits, including the preparation of zeolite frameworks [3,4,5] and compositions [6,7,8] that are challenging or even unattainable using classical approaches. It also provides solvent-free [9], quick crystallization [2,10,11,12], high yields [13,14], and cost-effective alternative OSDAs [15,16]. In addition, it provides alternative control over the particle size [13,17,18], morphology [13,19], adsorption [17], thermal properties [7,20], and phase purity [10,21]. Furthermore, by using this technique, mesopores can be created without the need for additional templating agents [13,22], metal clusters can be encapsulated [23], and zeolites with improved catalytic characteristics can be produced [8,13].

The **FAU** type of zeolite is one of the most studied frameworks as a starting material for IZT. This is due to its widespread commercial availability, easy synthesis, the broad range of framework Si/Al compositions, and its low-density framework favoring a wide variety of daughter phases.

The IZTs of **FAU** zeolites generally can be divided into inorganic- (OSDA-free, with or without seeds) and organic-based (OSDA-dependent with or without seeds) systems. The inorganic IZTs include examples such as **FAU**-**CHA** [9,17,24,25,26], **FAU**-**MFI** [24,27,28], **FAU**-**MER** [25], **FAU**-**ABW** [25], **FAU**-**ANA** [21,25], **FAU**-**STF** [24], **FAU**-**MTW** [24], FAU-**LEV** [2], **FAU**-**MAZ** [2], **FAU**-**LTL** [2], **FAU**-**GIS** [2], **FAU**-**MOR** [2,27], **FAU**-**FER** [2], **FAU**-**GME** [10,21,29], **FAU**-**HEU** [21], **FAU**-**BRE** [21], and **FAU**-**PHI** [21]. Organic (OSDA with or without seeds) IZTs comprise **FAU**-**CHA** [16,30,31], **FAU**-*BEA [13,32], **FAU**-**LEV** [5], **FAU**-**MTN** [33], **FAU**-**OFF** [34], **FAU**-**RUT** [35], **FAU**-**ERI** [36], **FAU**-**AFX** [37], **FAU**-**AEI** [38,39], **FAU**-**MFI** [23,38], **FAU**-**YFI** [3], **FAU**-**MWW** [40], **FAU**-**EMT** [39], **FAU**-**MAZ** [41], and **FAU**-**MSE** [42]. A common feature among these examples is the tendency of transformation to occur from a low- to a high-density framework and the need for an increased temperature to achieve the transformation.

A general comparison between the above-described IZTs shows that the OSDA-free approach combines several advantages that make it attractive for sustainable solutions in zeolite production. Benefits are associated with the reduction in the synthesis cost by the elimination of the OSDA molecules and the necessity for their post-synthesis removal related to high-temperature treatment. This thermal treatment may also affect the integrity of the zeolite structure, release harmful gases (CO_2_ and NO_x_), increase the number of preparation steps and the complexity of the synthesis protocol. Additionally, the use of OSDAs molecules contributes to waste generation and environmental pollution [43]. In a similar way, seed-free synthesis is simple and cost effective since it does not require the procurement or synthesis of additional seeds. Moreover, it minimizes the risk of contamination from foreign particles introduced by seeds, which is crucial for applications such as catalysis, adsorption, and ion exchange where impurities can affect performance.

In 1956, Barrer and Baynham reported the hydrothermal syntheses of the **EDI** type of zeolite [44] also known as Linde Type F, Barrer K-F (K_10_Al_10_Si_10_O_40_∙wH_2_O; w~8) [45,46], and Zeolite N (K_12_Al_10_Si_10_O_40_Cl_2_·5H_2_O) [47]. This zeolite is a potassium analogue of the barium aluminosilicate mineral Edingtonite [48]. As a trade name, Zeolite N is described as a MesoLite that finds applications in the removal of ammonia from water solutions [49,50,51,52]. Examples show its high capacity, high selectivity [49,50], and potential for ammonia removal in the presence of competing univalent and divalent cations [51]. Experiments have also demonstrated that MesoLite can be used as a soil conditioner that is superior to natural zeolite soil conditioners by significantly reducing the leaching of ammonia from sandy soil [53]. A clay mineral known as kaolin treated in an alkaline solution at 80–95 °C [53] or higher temperatures is usually used for the synthesis of MesoLite. Other routes to Zeolite N involve the hydrothermal treatment of zeolite 4A at 300 °C [47] or 130–175 °C [54] in solutions containing KOH and KCl. The classical gel-based hydrothermal synthesis of **EDI** zeolite is reported from systems containing K [55,56,57], Li [58], Li-Ba [59], Li-Cs [60,61], Cs-Na [62], Li-triethanolamine (TEA) [63], and tetramethylammonium (TMA)_2_O [64] at temperatures between 60 and 200 °C.

This work aims to demonstrate the transformation of **FAU** zeolite to **EDI** zeolite, a pioneering example of room-temperature IZTs, and exemplify an OSDA-free and seed-free conversion between zeolite frameworks lacking common composite building units (CBUs).

## 2. Results and Discussion

Since the thermodynamic stability and crystallization are closely related with the chemical potential of a system, it is reasonable to consider that the IZT is partly guided by this parameter. In this context, high concentrations of KOH solutions are hypothesized to activate novel IZTs. This consideration served as the principal rationale for selecting the synthesis conditions for IZT outlined in Table 1 (samples no. 1–16).

**Figure 1:**
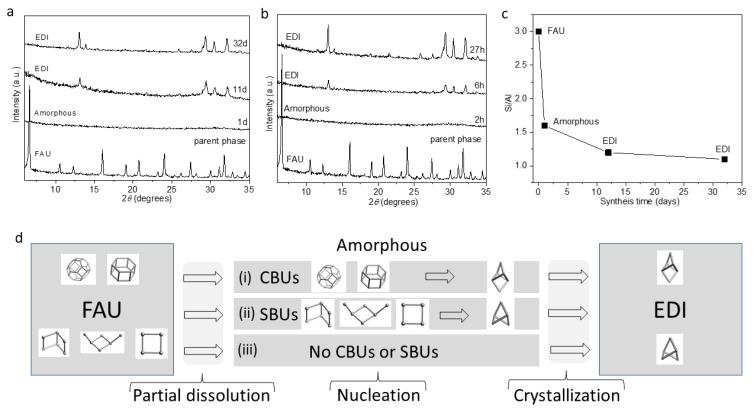
Powder XRD patterns following the (**a**) RT and (**b**) HT (60 °C) transformation of **FAU** zeolite to **EDI** zeolite at different times of synthesis. (**c**) The decrease in the Si/Al ratios in the run product over the synthesis time shows that independent of the lack of common CBUs, the **FAU** framework transforms to **EDI** zeolite. (**d**) A model assuming three possible compositions of the amorphous phase during the **FAU** to **EDI** transformation: (i) the CBUs (*sod* and *d6r*) of FAU that transform into the CBUs (*nat*) of EDI; (ii) the transformation of 4-2, 1-4-1, or 4 SBUs typical for **FAU** into 4 = 1 SBUs typical for the **EDI** framework; and (iii) a lack of CBUs and SBUs.

The XRD patterns (Figure 1a,b and Figures S1–S18) unambiguously demonstrate the successful transformation of an FAU zeolite into an **EDI** zeolite. The IZT of **FAU** (Si/Al = 3) to **EDI** (Si/Al = 1.1–1.3) occurs at room temperature (RT) (Figure 1a; samples no. 3, 7, 8, and 9) and under hydrothermal (HT) conditions (Figure 1b; samples no. 1 and 2). At RT, the EDI zeolite was detected after 11 d and under HT conditions (60 °C), the time for crystallization decreased to 6 h. These results suggest rates of nucleation and crystal growth that are accelerated by the higher temperature. In both instances, extending the synthesis time (RT: 32 d; HT: 27 h) leads to an increase in the relative crystallinity, with 55% at RT (Figure S19) and 65% under HT (Figure S20) conditions, indicating a continuous process of crystal maturation. In the RT synthesis, the crystallinity of EDI declines after 35 d, showing a 23% reduction compared to the crystallinity achieved after 32 d (Figure S19). The maximum yield of the samples obtained at RT (around 40%) is higher than that of those from the HT synthesis (around 30%). In both synthesis approaches, the Si/Al ratio remains slightly higher than 1 independent of the temperature and time of the IZT (Table 1).

Mechanistically, the **FAU**-to-**EDI** transformation includes the amorphization and partial dissolution of the parent phase followed by nucleation and crystallization. At RT, after 24 h in 20.43 M of a solution of KOH, the FAU framework collapses (Figure 1a). The same process of amorphization is accelerated at HT conditions and occurs for just 2 h (Figure 1b). The Si/Al ratio in the amorphous phase is 1.4–1.6 (samples no. 4, 5, and 6), which is notably lower than the Si/Al ratio of 3 in the parent crystalline phase. This negative correlation between the Si/Al ratio and the run product is observed throughout the process of IZT (Figure 1c) and indicates a dissolution mainly by desilication. This process is also evidenced by the progressively decreasing yield of the amorphous phase as the Si/Al ratio of the run product decreases (Figure S21). A similar mechanism of dissolution-mediated IZT has also been described by other researchers but under hydrothermal conditions [65].

Since the parent (**FAU**) and daughter (**EDI**) phases do not share common secondary or composite building units (https://www.iza-structure.org/databases/; accessed on 9 April 2024), it can be hypothesized that there are at least three routes for the transformation of the **FAU**-derived X-ray amorphous phase into **EDI** zeolite (Figure 1d): (i) the sodalite (*sod*) and the double six-ring (*d6r*) of the **FAU** structure transform to the natrolite (*nat*) CBU typical for **EDI** zeolite; (ii) secondary building units (SBUs) such as the 4-2, 1-4-1 or 4 of the **FAU** framework transform into 4 = 1 units typical for the **EDI** framework (https://www.iza-structure.org/databases/; accessed on 9 April 2024); or (iii) the transformation starts from the amorphous phase that lacks any secondary building units known for being in the parent and daughter phase. Similar IZTs between structures that do not share common CBUs rarely occur without using seeds or OSDAs [27].

**Figure 2:**
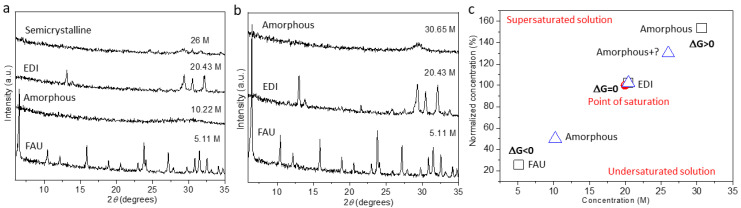
Dependence between the initial concentration of the KOH solution and the product of IZT performed under (**a**) HT conditions at 60 °C for 6 h and at (**b**) RT for 12 d. (**c**) Schematic relationship between the nucleation barrier and the initial concentration of KOH. Relative saturation level (%) of a solution compared to a reference concentration (M) showing the free energy deviations (ΔG > 0 and ΔG < 0) from the equilibrium solution (ΔG = 0; red solid circle—point of saturation) and the related phases obtained at RT (squares) for 12 d and under HT conditions (triangles) for 6 h.

Figure 2a,b shows the powder XRD patterns of the phases obtained via HT and RT IZTs, respectively, performed at different KOH concentrations. The high (26 M and 30.65 M) (samples no. 15 and 11) or relatively low (5.11 M and 10.22 M) (samples no. 10, 12, 13, 14, and 16) concentrations hinder the IZT to **EDI** zeolite at the selected time and temperature conditions. In the case of a high concentration, the run product is mainly in an amorphous phase and only weak and unindexed PXRD peaks suggest a competing process of crystallization. At low concentrations, the FAU structure remains preserved (5.11 M) (samples no. 10, 13, and 16) or transforms into an amorphous phase (10.22 M) (samples no 12 and 14). It is interesting to notice that at a low KOH concentration (5.11 M), the **FAU** structure contains a lower Si/Al ratio (2.1) (sample no. 13) than the parent **FAU** phase, pointing to an effective desilication without structural collapse. By plotting the relative saturation level versus the reference concentrations (Figure 2c) for the RT and HT conditions, it appears that the IZT is closely linked to the level of solution saturation (Figure 2c). A slightly supersaturated solution of KOH (20.43 M) (sample no. 7) creates conditions for crystallization, while the highly supersaturated one (30.65 M) (sample no. 11) inhibits the crystallization of **EDI** zeolite at RT. The undersaturated solution (5.11 M) (sample no. 10) is also not favorable for the **FAU**-to-**EDI** transformation at RT. Under HT conditions, both undersaturated and supersaturated solutions are not favorable for the crystallization of EDI zeolite for 6 h of synthesis. However, similar to the RT approach, the solution with a concentration close to the saturation point leads to EDI zeolite (Figure 2c). Considering the thermodynamics of a system, a solution in equilibrium is a saturated one and has Gibbs free energy (ΔG) that is close to zero. In the cases of supersaturated and undersaturated solutions, the ΔG is positive and negative, respectively. Appling these considerations to our system, it appears that **EDI** zeolite crystallizes from a solution that is close to equilibrium (Figure 2c). From the chemical point of view, the saturation levels influence the number of OH groups in the solution, thus affecting the dissolution level of the structural subunits. Accordingly, in an undersaturated solution, the parent phase is preserved while the saturated and supersaturated solutions result in a collapsed parent phase.

**Figure 3:**
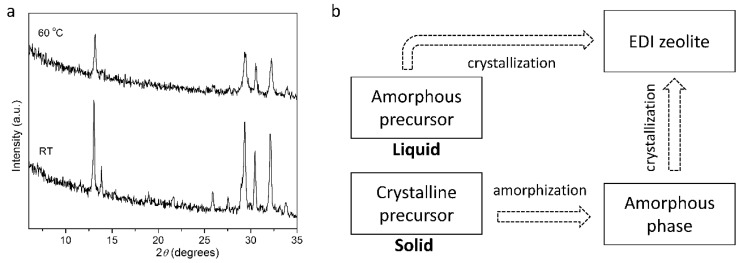
(**a**) Powder XRD patterns of **EDI** zeolite obtained from gel at room temperature (RT) for 32 d (sample no. 18) and at 60 °C for 22 h (sample no. 17) of synthesis. (**b**) Schematic presentation of the two pathways to **EDI** zeolite.

To gain further insight into the crystallization and to compare the zeolite obtained by the IZT and from the gel precursor, experimental conditions that led to the first RT synthesis of **EDI** zeolite by a classical method were developed. The powder XRD patterns in Figure 3a, Figure S7, S8, S15 and S16 show that **EDI** zeolite easily crystallizes at RT after 32 days (sample no. 18) or at 60 °C after 22 h (sample no. 17) from a synthesis gel with starting amounts of KOH and H_2_O like those of the IZT and SiO_2_/Al_2_O_3_ = 3.65–4.1. Unlike the IZT approach, the gel precursor has no structural memory of a parent crystalline phase and one can suggest that the amorphous phase transforming to **EDI** zeolite is not initially loaded with secondary building units, thus, their later formation is guided by the concentration of KOH at the given time and in the given temperature conditions. Compared to the RT IZT, in the classical synthesis, the Si/Al ratio (1.1–1.2) and the crystallinity of the **EDI** zeolite are similar. The crystallinity of the HT sample is 36% less than that of the **EDI** obtained using RT synthesis. This fact may be explained by the slower kinetics of crystallization at room temperature leading to a more organized structure. In comparison to other syntheses of potassium **EDI** zeolite with a high K_2_O/SiO_2_ ratio (9.95) [45,55], the recipe presented here does not contain more KOH (K_2_O/SiO_2_ = 8), which suggests that the combination of high alkalinity with proper sources of Al and Si drives the RT crystallization.

**Figure 4:**
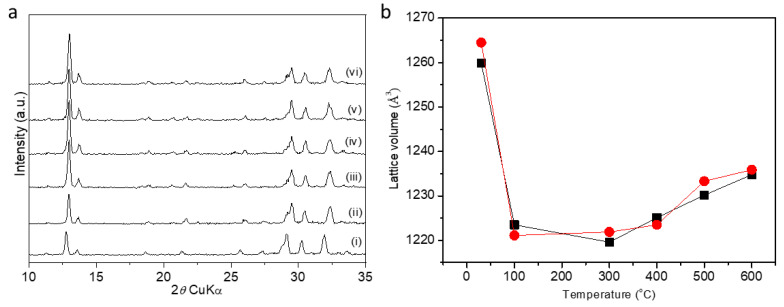
(**a**) Temperature-resolved powder XRD patterns of **EDI** zeolites obtained at (i) 30, (ii) 100, (iii) 300, (iv) 400, (v) 500, and (vi) 600 °C. (**b**) Change in the lattice volume refined in the space groups *I*222 (black squares) and *P*-42*m* (red circles) of **EDI** zeolite at different temperatures.

Figure 4 shows the powder XRD patterns and the related changes in the lattice volume of **EDI** zeolite (sample 8) between 30 and 600 °C refined by using a tetragonal (S.G. *P*-42*m*) and a orthorhombic (S.G. *I*222) lattice, respectively. When a tetragonal system is considered, the lattice volume shrinks by 3.5% at 100 °C. This step is followed by a lattice expansion of 1.2% at 600 °C. In the case of the assumed orthorhombic symmetry, the reduction in the cell volume by 3.3% finishes at 300 °C. In both cases (orthorhombic and tetragonal symmetry), the initial lattice shrinkage is due to dehydration of the structure [66]. Above 300 °C, gradual thermal expansion starts and at 600 °C, the lattice volume expands by 1.2%. Such a combination of negative followed by positive thermal expansion has been previously observed for the **FAU** type of zeolites [67].

Table 2 shows the refined (Figures S1–S16) lattice volumes of **EDI** types of zeolites synthesized at different conditions (Table 1). The refinement was performed considering the orthorhombic *I*222 and tetragonal *P*-42*m* space groups, respectively. In an attempt to clarify the right symmetry, a higher resolution pattern was collected and refined (Figures S17 and S18) but no reliable evidence was found favoring any of the lattices and both symmetries were included in the discussion. When comparing the mean differences of the refined volumes, it appears that the mean difference for the volumes extracted using space group *I*222 (≈8.1 Å^3^) is larger than that for the volumes extracted using space group *P*-42*m* (≈2.2 Å^3^). This indicates that, on average, the values in the orthorhombic lattice tend to deviate more from their mean value than the values in the tetragonal lattice, indicating a potentially higher error. However, the correct space group should be determined via the Rietveld method using high-resolution data. The same complexity also exists when comparing the changes in K/Si + Al ratios with the change in the lattice volume where no clear trend is observed.

**Figure 5:**
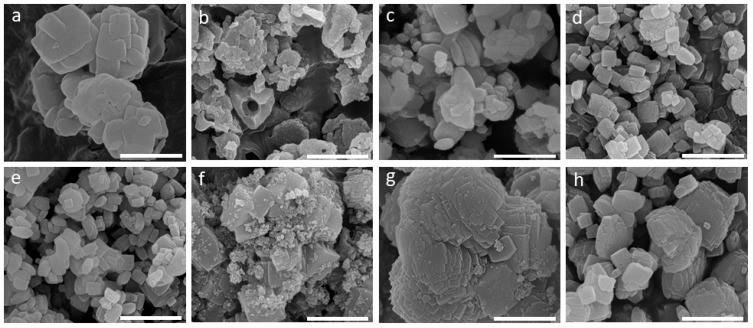
SEM images of the (**a**) parent **FAU** zeolite, the (**b**) amorphous phase obtained after 1 d, and the (**c**) **EDI** zeolite obtained after 12 d, (**d**) after 32 d, and (**e**) after 35 d of RT synthesis. (**f**) **EDI** zeolite obtained via IZT at 60 °C for 27 h. Classical synthesis of **EDI** zeolite (**g**) under HT conditions (60 °C) for 22 h and (**h**) at RT for 32 d of synthesis (bar = 1 µm).

The IZT approach to **EDI** zeolite offers control of the particle size and morphology and provides additional evidence of the process of the dissolution of the parent phase. After 1 d at room temperature in 20.43 M of a solution of KOH, the micron-sized (1000–1200 nm), rounded particles of **FAU** (Figure 5a) transform into smaller amorphous fragments with irregular shapes and clear signs (cavities) of dissolution (Figure 5b). As the synthesis progresses to 12 d, a mixture of nanoparticles (<100 nm) and larger (300–800 nm) intergrown faceted crystals appear (Figure 5c). The further increase in the crystallization time to 32 d leads to the formation of relatively uniform prismatic 100–400 nm crystals of **EDI** (Figure 4d), a size maintained up to 35 d (Figure 5e). The relative crystal size reduction from 12 d to 35 d of synthesis may be due to supersaturation changes and reversed Ostwald ripening dynamics. The **EDI** particles obtained via hydrothermal transformation from **FAU** zeolite appear as aggregates (>1000 nm) composed of nanocrystals (<100 nm) decorating larger (300–700 nm) faceted crystals (Figure 5f). The particles resulting from a hydrothermal reaction of a synthesis gel show larger (2000–3000 nm) and less-uniform intergrown crystals (Figure 5g). Conversely, when obtained from the same gel at room temperature, less-aggregated and smaller-sized (100–1000 nm) prismatic crystals emerge (Figure 5h). Regardless of the system (IZT or gel), these observations consistently highlight that the slower RT crystallization favors the formation of generally more uniform and less-aggregated crystals over larger, more-aggregated crystals which are more likely to form during a faster crystallization. A plausible explanation for this result is the low nucleation rate and the lower supersaturation at RT conditions.

**Figure 6:**
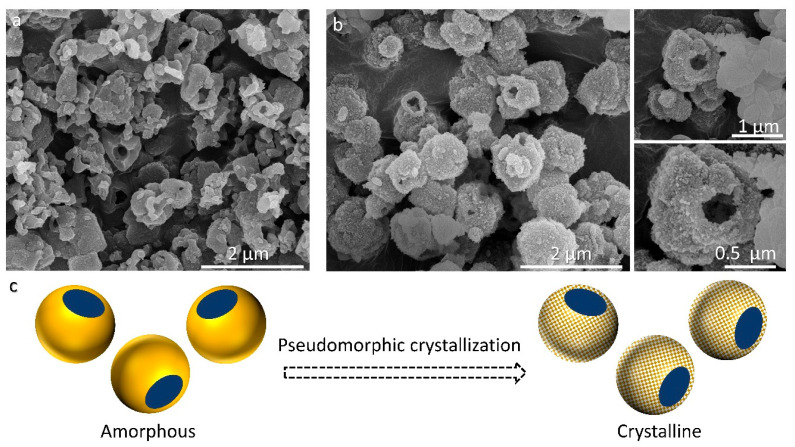
SEM images showing the peseudomorphic transformation of the (**a**) amorphous phase with cavities and hollow particles to (**b**) **EDI** zeolite with a similar morphology obtained at RT for 1 d (sample no. 4) and 11 d (sample no. 9), respectively. (**c**) Schematic model of the observed pseudomorphic crystallization.

Surprisingly, the IZT performed for 11 d resulted in hollow submicron particles composed of nanoparticles resembling the amorphous particles obtained after 1 d of synthesis (Figure 6a,b). This observation represents a crystallization that involves a pseudomorphic transformation of an amorphous phase (Figure 6c). This suggestion is also supported by the PXRD pattern (Figure 1a) where the peaks of **EDI** zeolite coexist with an amorphous hump. Another conclusion of this observation is that the nucleation and the crystallization of EDI particles occurs within an amorphous phase by mimicking the pre-existing morphology. Although a recent example has shown the crystallization of hierarchical **BEA** zeolite from **FAU** zeolite using OSDAs [13], to the best of our knowledge, hierarchical structures of zeolites obtained via pseudomorphic crystallization during IZT have never been reported. It is important to note that the hierarchical structure here describes the organization of particles, not of porosity. A comparison of the hollow particles to other **EDI** morphologies obtained under varying time and temperature conditions (Figure 5) reveals that various metastable morphologies exist within the IZT.

Considering the results, the synthesis of **EDI** zeolite from **FAU** zeolite offers several advantages over the gel synthesis. **FAU** zeolite is widely available, and is a thermally and chemically stable eco-friendly material whose use as a silica and alumina source reduces the producing steps by eliminating the need for different starting materials, storage in controlled conditions, dissolution and complex mixing. This can lead to cost savings and increased sustainability in **EDI** zeolite synthesis. Furthermore, the **FAU**–**EDI** transformation allows for the selective development of specific particle morphologies that are not achievable through classical gel synthesis alone. In this case, the **FAU**–**EDI** transformation provides access to hollow hierarchical particles. This crystal morphology selectivity holds potential for the synthesis of **EDI** zeolite with tailored properties. Finally, the **FAU**–**EDI** transformation offers a new system for fundamental studies of nucleation and crystal growth mechanisms in zeolites.

## 3. Experimental

*Materials.* SiO_2_ (Ludox AS-40, colloidal silica; Sigma-Aldrich, St. Louis, MI, USA), SiO_2_ (fumed silica; Sigma-Aldrich), NaAlO_2_ (Al_2_O_3_ 50–56%; Na_2_O 37–45%; Sigma-Aldrich), NaOH (≥98%, Sigma-Aldrich), and KOH (90%, Sigma-Aldrich) were used.

*Synthesis of the parent FAU zeolite.* Synthesis was performed following procedures similar to those previously reported [68,69]. Namely, a solution of 3.0 g of sodium aluminate, 2.2 g of NaOH, and 22.8 g of distilled water was added to 22.5 g of colloidal silica. The obtained gel, with a molar composition of 4.41 NaOH: 2.93 NaAlO_2_: 12 SiO_2_: 161.5 H_2_O, was stirred virtuously, aged at room temperature for 1 d, then transferred in a polypropylene bottle (60 mL) and heated for 3 d at 100 °C. After the synthesis, the run product was filtered and washed several times with distilled water in order to remove the mother liquid and other impurities. This procedure for sample recovery was applied to all syntheses.

*Interzeolite transformation*. The IZTs were performed following the conditions listed in Table 1. In a typical synthesis, 0.5 g of the **FAU** was placed in a KOH water solution (3.9 g of distilled water) with different concentrations. The reaction was performed in a closed polypropylene bottle (30 mL). After the selected synthesis times and temperatures (a room temperature of 24 ± 1 °C or 60 °C), the run product was filtered, washed several times with distilled water, and dried at 40 °C. The yield was determined by comparing the weight of the initial parent phase with the weight of the solid product of the synthesis. The saturation level of the solution was calculated considering the solubility of KOH at 25 °C (approximately 121 g per 100 mL of water).

*Classical synthesis.* In a typical synthesis, 0.3 g of fumed silica was dissolved in a solution of 4.47 g of KOH and 3 g of distilled water. Subsequently, a solution of 0.2 g of NaAlO_2_ and 0.9 g of distilled water was added to the above mixture. The obtained white gel with a composition 18.38 K_2_O: 1.13 NaAlO_2_: 2.3 SiO_2_: 100 H_2_O was homogenized and poured into a polyethylene bottle. In two separate experiments, crystallization was performed at room temperature for 32 d and at 60 °C for 22 h.

*Characterization.* The powder X-ray diffraction (XRD) patterns were collected using diffractometer Bruker D8 Discover working with copper radiation (step: 0.04°, time per step: 0.2 s) and a LynxEye position-sensitive detector. The in situ high-temperature XRD patterns were collected using an Anton Paar HTK1200 chamber and a heating rate of 0.5 °C/s. The phase identification was performed by the International Center for Diffraction Data database (PDF: 087-2476 and 038-0216 for **EDI**; 076-0591 for **FAU**) integrated into EVA software (Bruker AXS). The unit cell parameters were refined by the Le Bail method using TOPAS-3 (Bruker AXS) software using an orthorhombic lattice with space group *I*222 [47] and a tetragonal lattice with space group *P*-42*m* [70]. Considering that **EDI** zeolite can crystallize in both symmetries, a higher resolution powder XRD pattern (step 0.01°, 7–90 2*θ*°) was also recorded and refined in an attempt to elucidate the right symmetry. The crystallinity of the EDI zeolite was estimated by comparing the integrated intensity of the peaks between 28 and 32.5 2*θ*°. The scanning electron microscopy (SEM) micrographs and the energy dispersive spectroscopy (EDS) chemical analysis were performed on a NanoSEM-FEI Nova 200 equipped with EDAX—Pegasus X4M.

## 4. Conclusions

In summary, this study communicates the following findings: (1) the first interzeolite transformation from **FAU** zeolite to **EDI** zeolite, (2) the KOH concentration of the initial solution guides the IZT, (3) the first room-temperature synthesis of **EDI** zeolite using a classical gel method, and (4) the crystallization of hollow **EDI** particles. Finally, the room temperature approach to **EDI** zeolite opens up a potential for other low-temperature interzeolite transformations, thus offering an energy-efficient method for zeolite synthesis.

## Figures and Tables

**Table 1:** Synthesis conditions and related results.

No.	Si & Al Source	Conc. (M)	KOH/FAU (wt.)	H_2_O/FAU (wt.)	Time	T °C	Product	Si/Al
1	**FAU**	20.43	8.94	7.8	27 h	60	EDI	1.1
2	**FAU**	20.43	8.94	7.8	6 h	60	EDI	1.2
3	**FAU**	20.43	8.94	7.8	35 d	RT	EDI	1.1
4	**FAU**	20.43	8.94	7.8	24 h	RT	am.	1.6
5	**FAU**	20.43	8.94	7.8	2 h	60	am.	1.4
6	**FAU**	20.43	8.94	7.8	4 h	60	am. + ?	1.4
7	**FAU**	20.43	8.94	7.8	12 d	RT	EDI	1.2
8	**FAU**	20.43	8.94	7.8	32 d	RT	EDI	1.1
9	**FAU**	20.43	8.94	7.8	11 d	RT	EDI	1.3
10	**FAU**	5.11	2.24	7.8	12 d	RT	FAU	N/A
11	**FAU**	30.65	13.5	7.8	12 d	RT	am.	N/A
12	**FAU**	10.22	4.47	7.8	6 h	60	am.	N/A
13	**FAU**	5.11	2.24	7.8	6 h	60	FAU	2.1
14	**FAU**	10.22	4.47	7.8	4 h	60	am.	N/A
15	**FAU**	26	11.38	7.8	6 h	60	am. + ?	N/A
16	**FAU**	5.11	2.24	7.8	9 d	RT	FAU	N/A
17	SiO_2_ & NaAlO_2_	Gel synthesis			22 h	60	EDI	1.1
18	SiO_2_ & NaAlO_2_	Gel synthesis			32 d	RT	EDI	1.2

Si/Al—determined by EDS; am.—amorphous; RT—room temperature; SiO_2_—fumed silica; Conc.—molar concentration; ?—contain unindexed peaks.

**Table 2:** Refined lattice volumes in space groups *I*222 and *P*-42*m* and chemical compositions of **EDI** zeolites synthesized at different conditions.

Sample No.	K/Al + Si	*V* (Å^3^) S.G. *I*222	*V* (Å^3^) S.G. *P*-42*m*
1	0.5	1277.9 (1)	1276.1 (2)
2	0.4	1281.8 (8)	1281.8 (5)
3	0.7	1277.3 (4)	1274.4 (1)
7	0.6	1276.6 (1)	1277.8 (8)
8	0.4	1276.8 (1)	1277.6 (2)
9	0.4	1241.5 (1)	1276.1 (1)
17	0.9	1283.0 (4)	1280.9 (1)
18	0.6	1276.5 (1)	1274.2 (2)

## Data Availability

The data presented in this study are available in this article.

## Supplementary Materials

The following supporting information can be downloaded at: https://www.mdpi.com/article/10.3390/molecules29081744/s1.
