# Supplementary material for: Interzeolite Transformation from FAU-to-EDI Type of Zeolite
Source: Molecules. 2024 Apr 11;29(8):1744. doi: 10.3390/molecules29081744 (PMC11052343; doi:10.3390/molecules29081744)
Supplement: Supplementary file 1 [file molecules-29-01744-s001.zip › molecules-2926298-supplementary.pdf]

Supporting Information

Interzeolite Transformation from FAU-to-EDI type of zeolite

Stanislav Ferdov

Physics Centre of Minho and Porto Universities (CF-UM-UP), University of Minho, 4800-058 Guimarães, Portugal

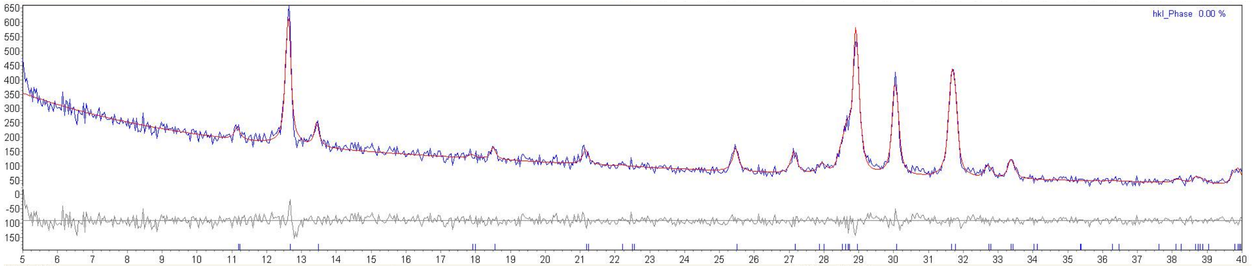

Figure S1. Sample no. 1 (S.G. I222)

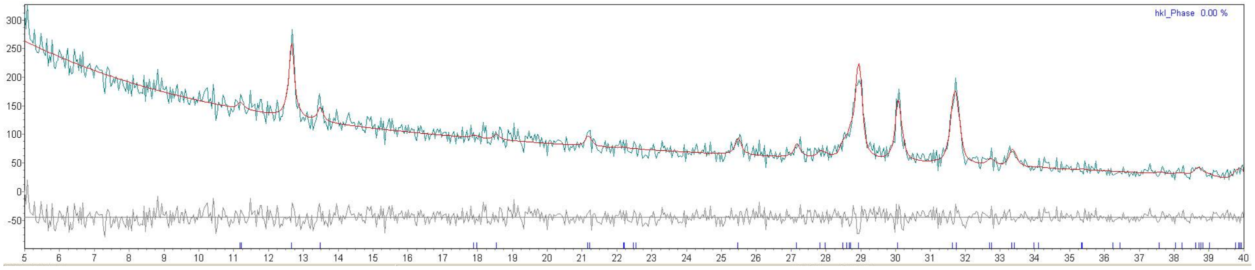

Figure S2. Sample no. 2 (S.G. I222).

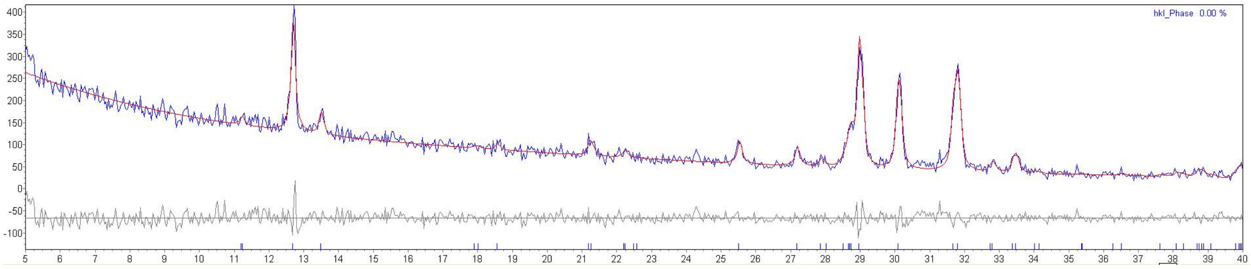

Figure S3. Sample no. 3 (S.G. I222).

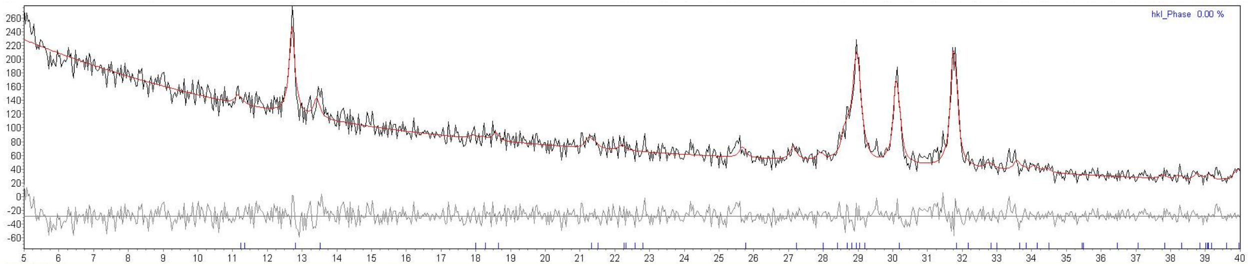

Figure S4. Sample no. 7 (S.G. I222).

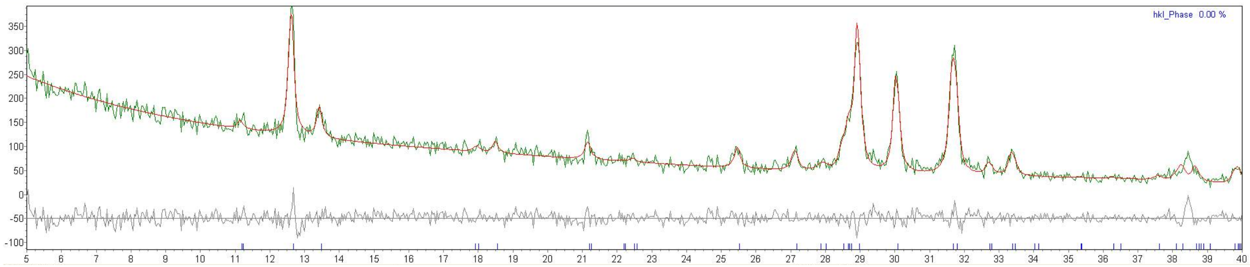

Figure S5. Sample no. 8 (S.G. I222).

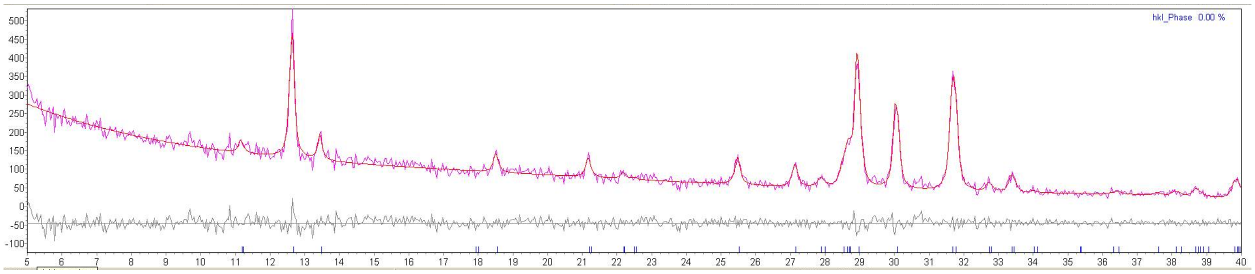

Figure S6. Sample no. 9 (S.G. I222).

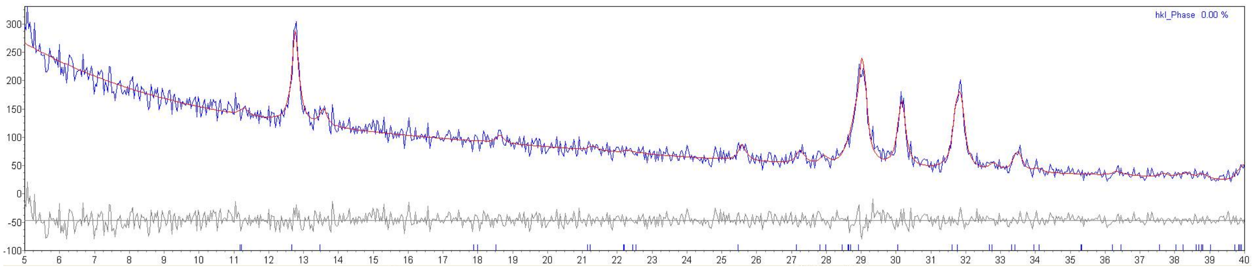

Figure S7. Sample no. 17 (S.G. I222).

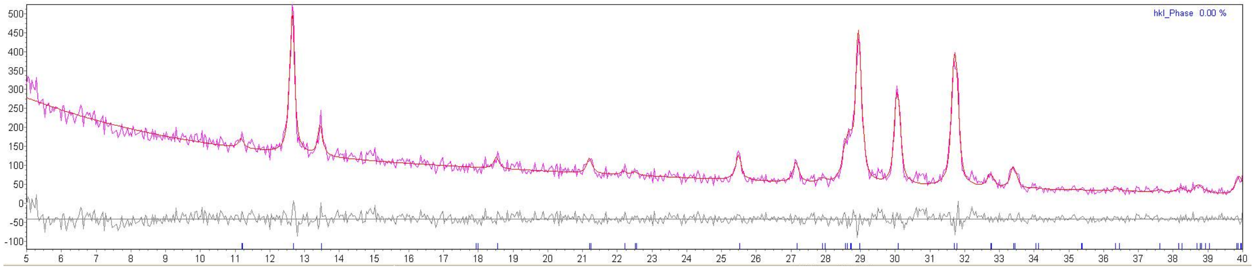

Figure S8. Sample no. 18 (S.G. I222).

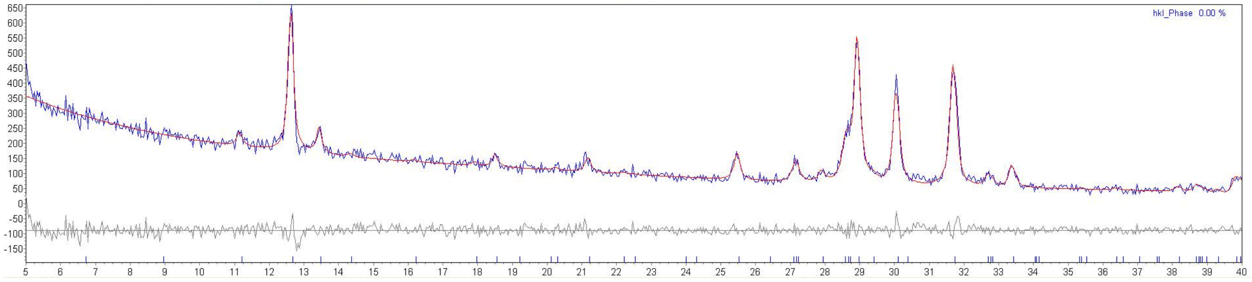

Figure S9. Sample no. 1 (S.G. P-42m).

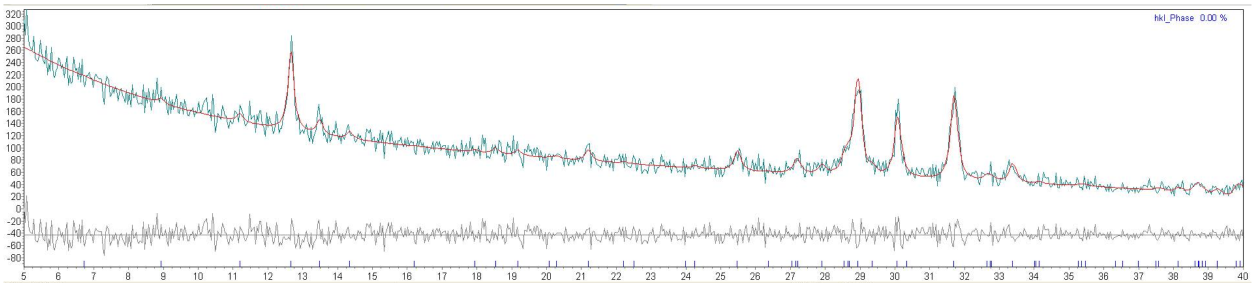

Figure S10. Sample no. 2 (S.G. P-42m).

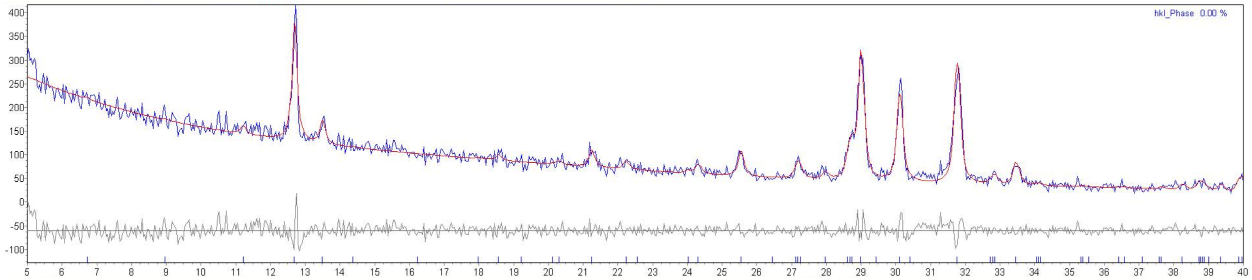

Figure S11. Sample no. 3 (S.G. P-42m).

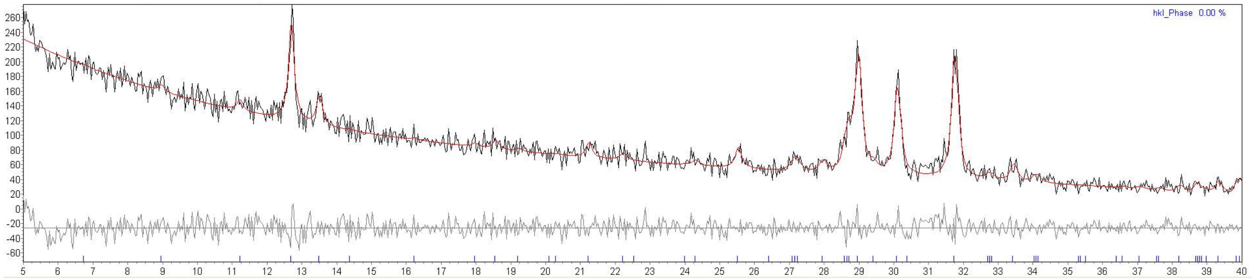

Figure S12. Sample no. 7 (S.G. P-42m).

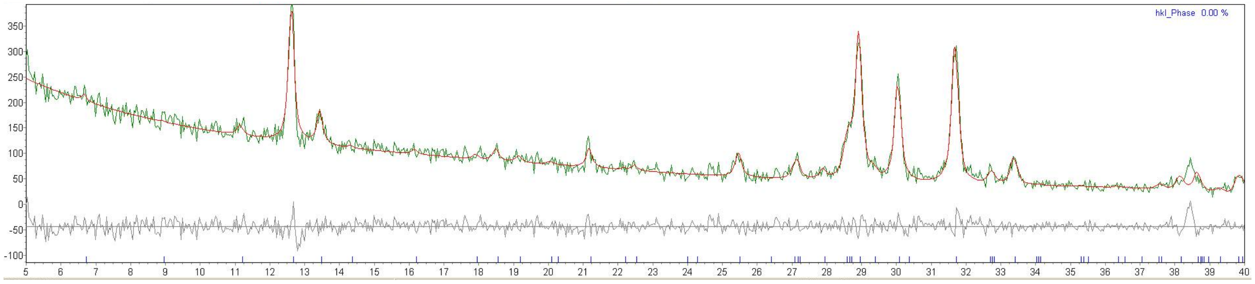

Figure S13. Sample no. 8 (S.G. P-42m).

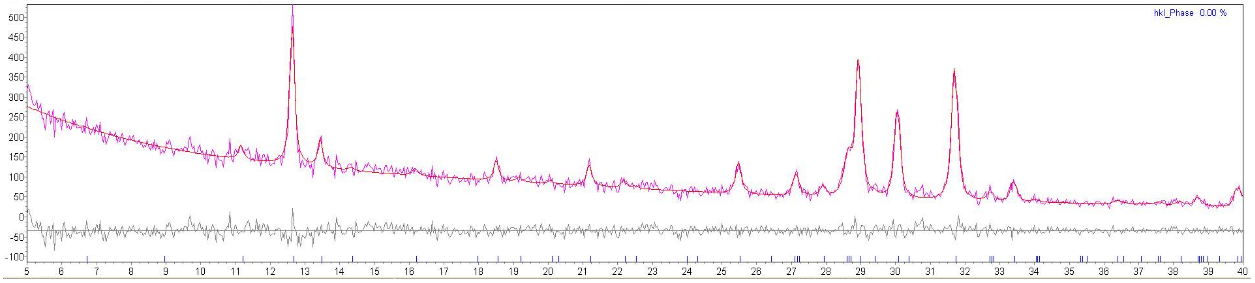

Figure S14. Sample no. 9 (S.G. P-42m).

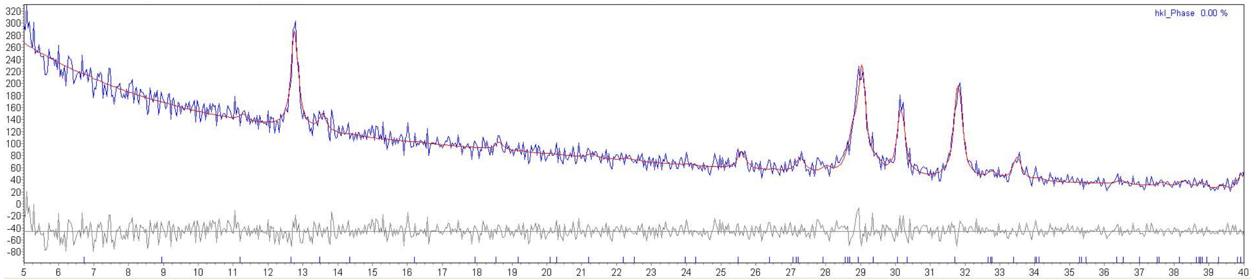

Figure S15. Sample no. 17 (S.G. P-42m).

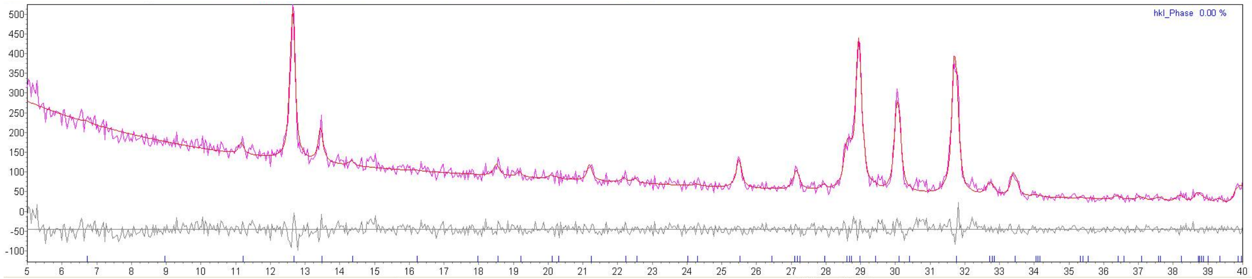

Figure S16. Sample no. 18 (S.G. P-42m).

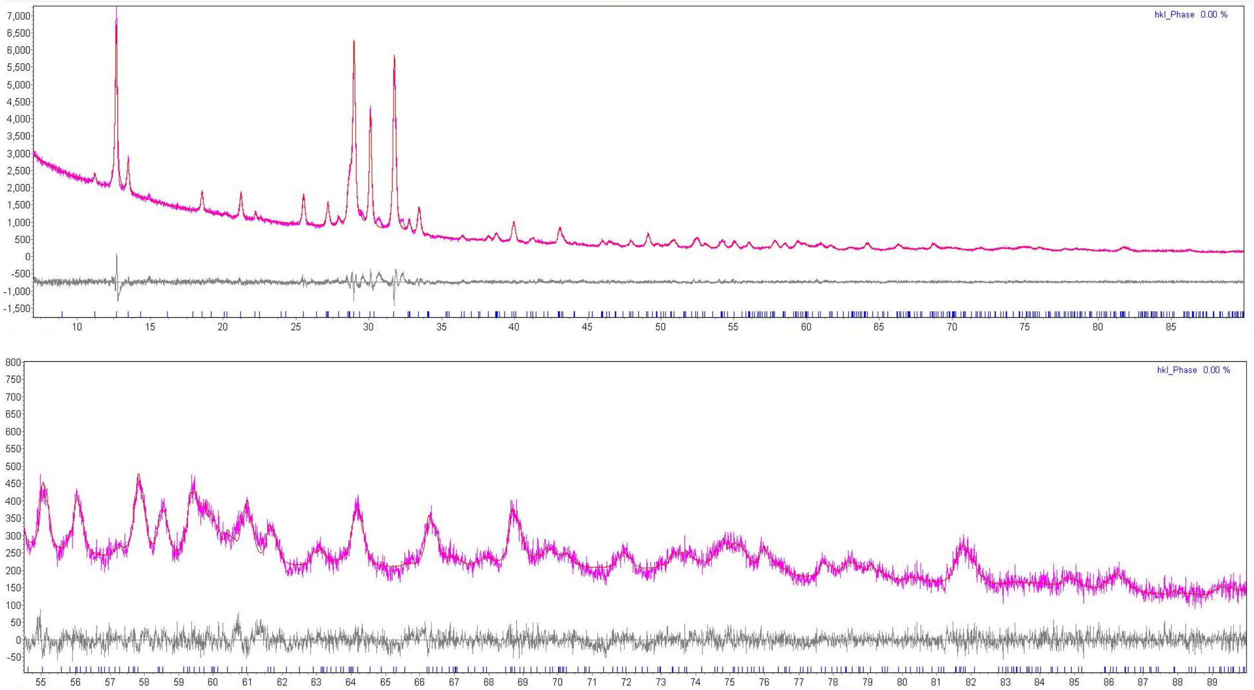

Figure S17. Le Bail fit of high-resolution PXRD data using space group P-42m (sample no. 8) Rwp: 5.49.

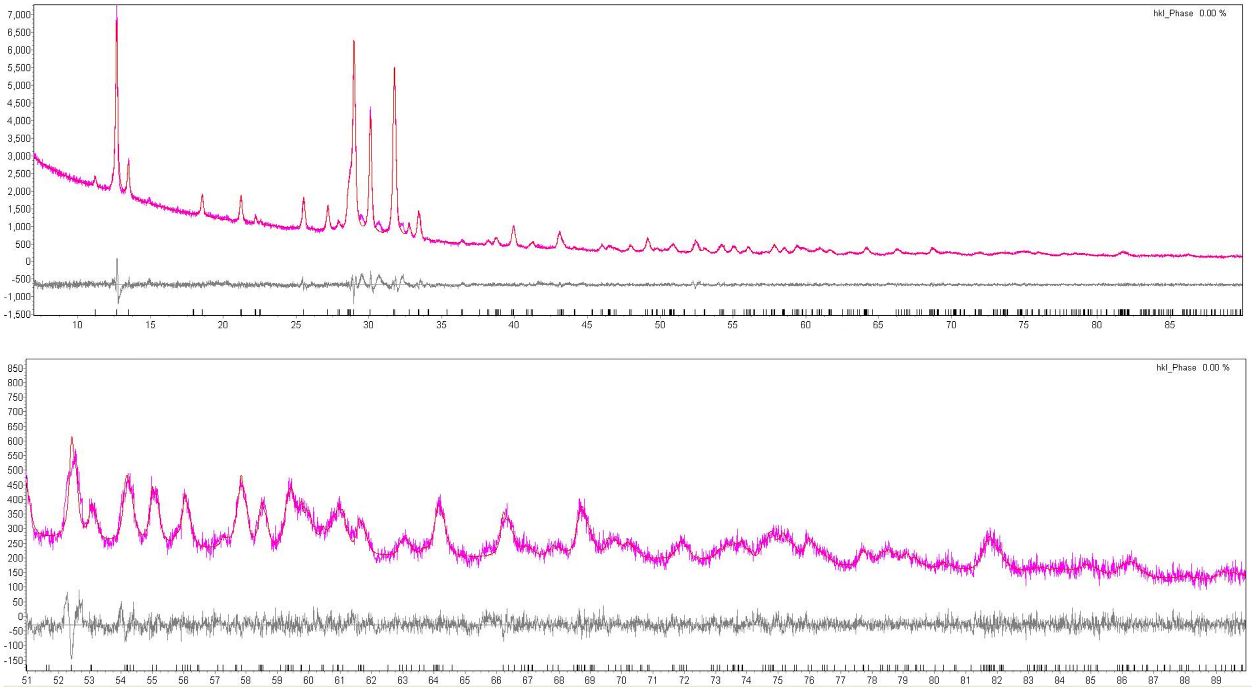

Figure S18. Le Bail fit of high-resolution PXRD data using space group I222 (sample no. 8) Rwp: 5.48.

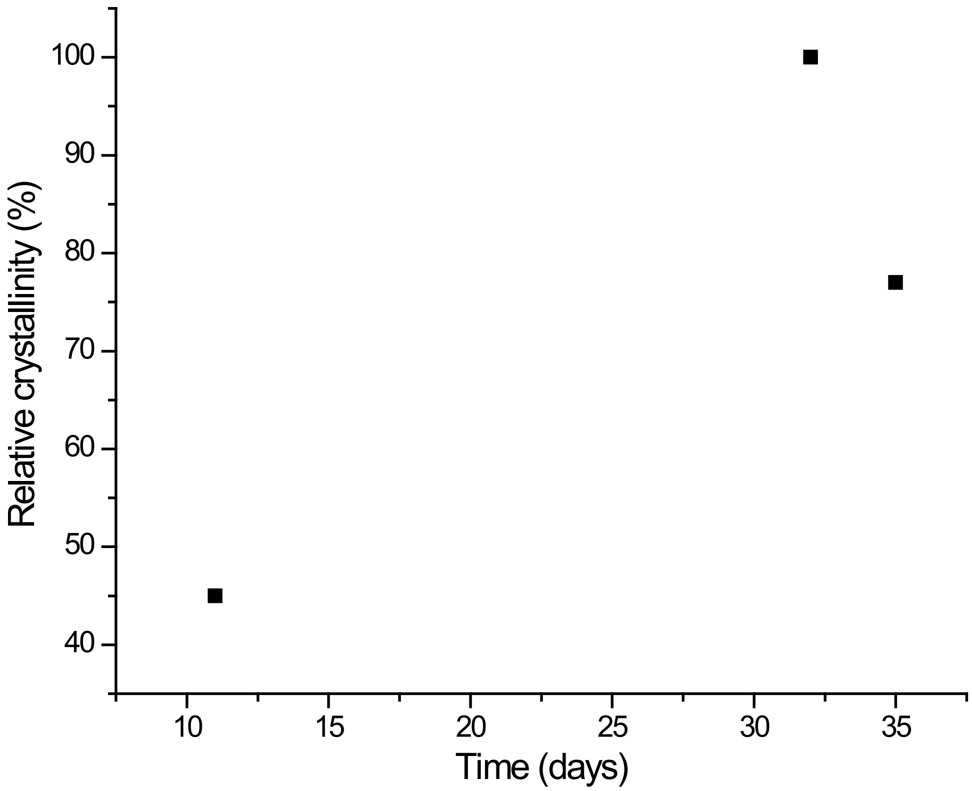

Figure S19. Change of the relative crystallinity of EDI zeolite during the EDI to FAU IZT at RT.

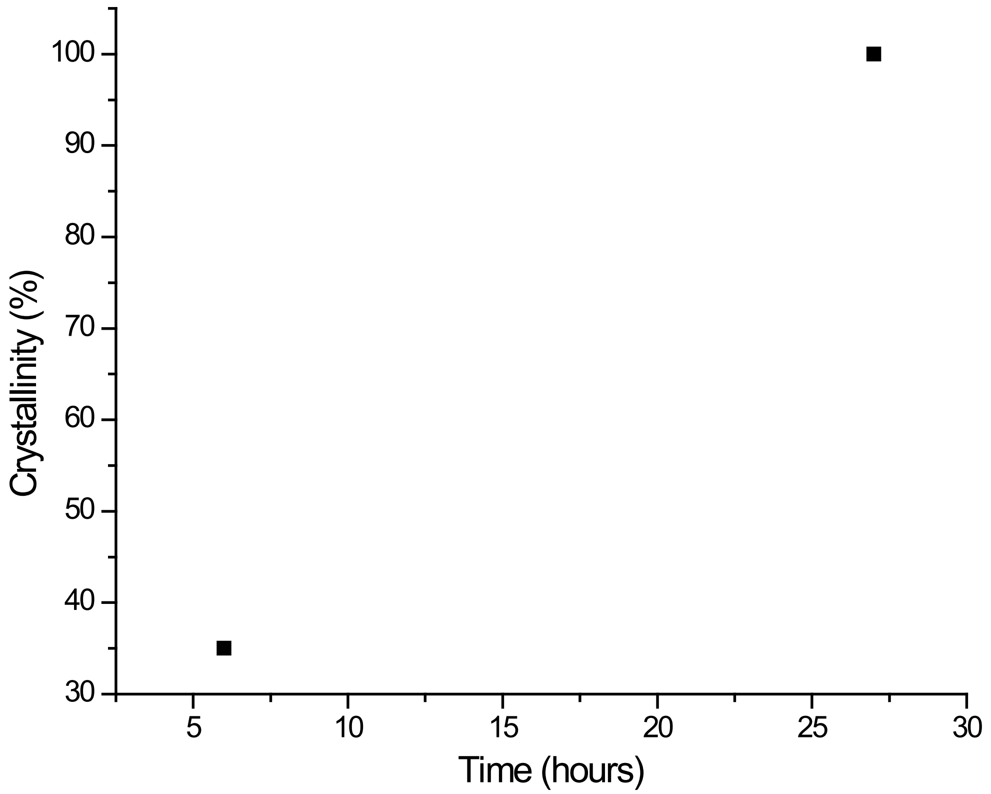

Figure S20. Change of the relative crystallinity of EDI zeolite during the EDI to FAU IZT at HT conditions.

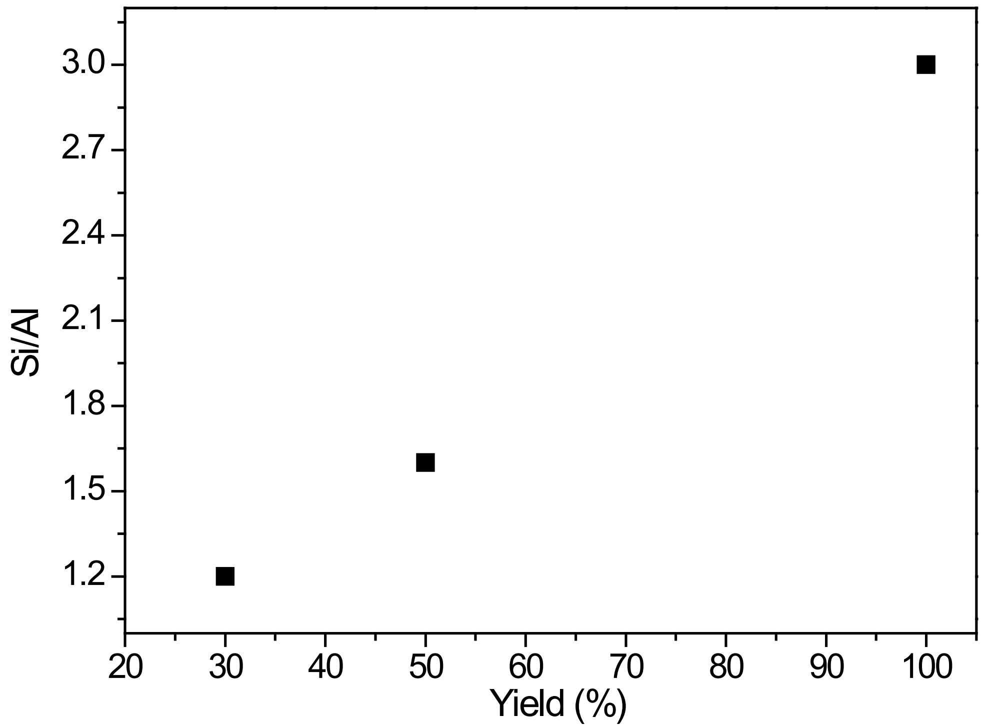

Figure S21. Representative example of a correlation between the Si/Al ratio and the yield of the run product.
